# Long-term prognostic implications of risk factors associated with tumor size: a case study of women regularly attending screening

**DOI:** 10.1186/s13058-018-0962-6

**Published:** 2018-04-18

**Authors:** Fredrik Strand, Keith Humphreys, Johanna Holm, Mikael Eriksson, Sven Törnberg, Per Hall, Edward Azavedo, Kamila Czene

**Affiliations:** 1 Department of Medical Epidemiology and Biostatistics, Karolinska Institutet, Nobels Väg 12A, 171 77 Stockholm, Sweden; 2 Thoracic Radiology, Karolinska University Hospital, Stockholm, Sweden; 3 Department of Cancer Screening, Stockholm-Gotland Regional Cancer Centre, Stockholm, Sweden; 4 Department of Oncology, South General Hospital, Stockholm, Sweden; 5 Department of Molecular Medicine and Surgery, Karolinska Institutet, Stockholm, Sweden

**Keywords:** Breast cancer, Early detection, Screening, Body mass index, Mammographic density, Prognosis

## Abstract

**Background:**

Breast cancer prognosis is strongly associated with tumor size at diagnosis. We aimed to identify factors associated with diagnosis of large (> 2 cm) compared to small tumors, and to examine implications for long-term prognosis.

**Methods:**

We examined 2012 women with invasive breast cancer, of whom 1466 had screen-detected and 546 interval cancers that were incident between 2001 and 2008 in a population-based screening cohort, and followed them to 31 December 2015. Body mass index (BMI) was ascertained after diagnosis at the time of study enrollment during 2009. PD was measured based on the contralateral mammogram within 3 years before diagnosis. We used multiple logistic regression modeling to examine the association between tumor size and body mass index (BMI), mammographic percent density (PD), or hormonal and genetic risk factors. Associations between the identified risk factors and, in turn, the outcomes of local recurrence, distant metastases, and death (153 events in total) in women with breast cancer were examined using Cox regression. Analyses were carried out according to mode of detection.

**Results:**

BMI and PD were the only factors associated with tumor size at diagnosis. For BMI (≥25 vs. < 25 kg/m^2^), the multiple adjusted odds ratios (OR) were 1.37 (95% CI 1.02–1.83) and 2.12 (95% CI 1.41–3.18), for screen-detected and interval cancers, respectively. For PD (≥20 vs. < 20%), the corresponding ORs were 1.72 (95% CI 1.29–2.30) and 0.60 (95% CI 0.40–0.90). Among women with interval cancers, those with high BMI had worse prognosis than women with low BMI (hazard ratio 1.70; 95% CI 1.04–2.77), but PD was not associated with the hazard rate. Among screen-detected cancers, neither BMI nor PD was associated with the hazard rate.

**Conclusions:**

In conclusion, high BMI was associated with the risk of having a tumor larger than 2 cm at diagnosis. Among women with interval cancer, high BMI was associated with worse prognosis. We believe that women with high BMI should be especially encouraged to attend screening.

**Electronic supplementary material:**

The online version of this article (10.1186/s13058-018-0962-6) contains supplementary material, which is available to authorized users.

## Background

Breast cancer screening has been estimated to reduce breast cancer mortality by around 30%; 23% among those invited and 40% among those attending [1]. In a recent study, Welch et al. argued that after the introduction of population-based screening, the number of tumors that were ≥ 2.0 cm in size had not decreased as much as expected [2]. It has been estimated that the 5-year survival rate is 79.8 and 93.1% in patients with tumors that are 2.0 to 4.9 cm and < 2.0 cm in size, respectively [3]. Thus, identifying women at risk of being diagnosed with a large tumor may be the best approach to further reduce breast cancer mortality.

Breast cancers are detected either through mammographic screening or by clinical symptoms. Among women who regularly attend screening and are diagnosed with breast cancer, about 30% are interval cancers [4], a term used for cancers detected clinically in the time interval after a negative screen and before the next planned screen. Clinical detection is in the majority of cases initiated by the woman herself, or her gynecologist, palpating a mass in the breast [5]. Patient characteristics hindering mammographic screen detection may well differ from those hindering clinical detection.

We aimed to understand the risk factors that predispose women to being diagnosed with a tumor > 2 cm in size. Since our long-term goal is to individualize the screening process we focused on risk factors that could be measured before diagnosis. We stratified analyses by detection mode to understand how each risk factor might be related to clinical and mammographic detectability. We also examined how the identified risk factors influence long-term prognosis and relate to tumor molecular subtypes.

## Methods

The study was based on women diagnosed with invasive breast cancer between 2001 and 2008 in a Swedish population-based screening cohort of the Stockholm-Gotland region. The case cohort, Libro-1, has been described in detail previously [6]. During the time period 2001 to 2005, the screening program in Stockholm involved inviting all women between age 50 and 69 years to screening every 24 months. From 2005 onwards screening was gradually extended to women between age 40 and 49 years. Our study population consists only of women 50 years of age or older. The average screening participation rate was 70%, the recall rate was 3%, and the cancer detection rate was 0.5%. Each screening involved a radiology nurse asking the woman about any breast symptoms and acquiring an analog film mammogram. Screening did not involve palpation by medical professionals, i.e., clinical breast examinations. After image acquisition, the mammograms were independently reviewed by two radiologists, and if either of them noted a suspicious finding they went on to reach a consensus decision. For further details, please see Lind et al. [7]. Since risk factors might differ depending on menopausal status, we included only women more than 50 years of age at diagnosis. Women were excluded if information was missing on tumor size or detection mode. The final study sample consisted of 2012 women. The regional ethical review board granted ethical approval and all participants gave written informed consent.

Large tumors were defined as tumors where the greatest dimension exceeded 2.0 cm at histopathologic assessment, a size cutoff also used in the TNM staging system [8]. For women with multiple tumor foci, the largest invasive focus was measured.

Detection mode was ascertained using the population-based Screening Register at the Regional Cancer Centre Stockholm-Gotland. The cancer was defined as screen-detected if the woman was recalled from screening and diagnosed during the following diagnostic work-up. The cancer was defined as interval cancer if it was clinically diagnosed within a normal screening interval after a negative screening. For women in the study, the regular time interval between screenings was 24 months. Analog film mammograms were collected from radiology departments and digitized with an Array 2905HD Laser Film Digitizer (Array Corp, Tokyo, Japan). Mammographic percent density (PD) represents the proportion of the breast area in the mammogram that is covered by dense breast tissue. It was measured on one mammogram per woman based on the contralateral mediolateral oblique view, from up to 3 years before and including time of diagnosis, using an automated method previously described [9]. Briefly, the method mimics the gold standard PD measurement method Cumulus [10], which is based on a semi-automated thresholding procedure. Data on hormonal and reproductive history and anthropometric risk factors were collected from web and paper questionnaires from the time of study enrollment in 2009, which was after diagnosis. The median time between diagnosis and enrollment was 4.8 years (interquartile range 3.0–6.6 years). Body mass index (BMI) was calculated from self-reported height and weight.

Data on tumor characteristics were obtained through linkage with the Swedish Cancer Register and the Breast Cancer Quality Register at the Regional Cancer Centre Stockholm-Gotland using the Swedish personal identity numbers. Missing data on molecular markers were retrieved from medical records. The surrogate molecular subtype (molecular subtype) was defined based on the consensus of the 13th St Gallen International Breast Cancer Conference (2013) Expert Panel [11]. The tumor was assigned the luminal subtype if it was estrogen receptor (ER) or progesterone receptor (PR) positive (or both), and if it was human epidermal growth factor receptor 2 (Her2) negative; if in addition Ki-67 expression was < 14% we assigned it the luminal A subtype, and if Ki-67 was ≥ 14% we assigned it the luminal B subtype. The tumor was assigned the Her2-overexpressing subtype if it was ER and PR negative and Her2 positive, and the basal-like subtype if it was ER, PR, and Her2 negative. Percent ER and PR staining were dichotomized into positive or negative status using a cutoff ≥10%. Her2 was considered negative if protein expression was 0 or 1+, or was higher with no confirmed gene amplification by fluorescence in-situ hybridization (FISH), and positive if FISH showed gene amplification.

Genotyping was based on blood samples, collected at the time of study enrollment, and performed on a custom Illumina iSelect Array (iCOGS) comprising 211,155 single nucleotide polymorphisms (SNPs). Definition of 77 breast cancer SNPs was based primarily on variants reported to be associated at a genome-wide level (*p* < 5 × 10^− 8^) by COGS or previous publications with either breast cancer overall or different ER subtypes of cancer [12]. The polygenic risk scores for the women in our study were then calculated by summing the number of alleles for each of these 77 SNPs, weighted by the effect sizes reported by Mavaddat et al. [12] (Additional file 1: Table S1).

For long-term follow up, the outcome of interest was defined as the first occurrence of local recurrence, distant metastasis, or death due to breast cancer (collectively “breast-cancer events”). Information on the date of local recurrence and distant metastasis was retrieved from the Breast Cancer Quality Register at the Regional Cancer Centre Stockholm-Gotland [13]. Date and cause of death were retrieved from the Cause of Death Register [14]. Information on dates of emigration was retrieved from the Swedish Emigration Register. Patients were followed from the date of diagnosis until a breast-cancer event, death by other cause, emigration, or end of study period (31 December 2015), whichever came first.

### Methods - statistical analysis

We fitted logistic regression models with dichotomized tumor size as the outcome and potential risk factors as covariates. The potential risk factors were age at diagnosis, BMI, education level, PD, age at menarche, ever use of oral contraceptives, nulliparity, age at first child birth, ever use of hormone replacement therapy, first-degree family history of breast cancer, and polygenic risk score. Odds ratios (ORs) were estimated both crudely and after multiple adjustments. Then, stratified by detection mode, the associations between the identified risk factors and tumor size were examined by logistic regression modeling. PD and BMI were dichotomized for analysis; the cut point for PD was 20% and the cut point for BMI was 25 kg/m^2^. Linear associations between these two predictors and tumor size as a continuous variable were examined by linear regression modeling. We constructed a figure describing the model-predicted probabilities of having a large tumor based on an age-adjusted logistic regression model using PD and BMI as continuous variables. As there was a delay between diagnosis and anthropometric risk factor ascertainment that differed between women, we conducted sensitivity analysis of the association between BMI and tumor size, using three separate regression models depending on the length of the delay (less than 3 years, from 3 years to less than 6 years, or 6 years or more). To understand the long-term prognostic implications, we fitted a multiple adjusted Cox regression model to study time to breast-cancer event as a function of tumor size and the identified risk factors. The proportional hazards assumption was checked by studying the Schoenfeld residuals (no violation was observed). Potential bias due to differential survival from diagnosis to study entry was examined by introducing a term for the time between diagnosis and study entry. Finally, we fitted multinomial logistic regression models, overall and separately within each detection mode, to estimate the relative risk ratio (RRR) for the associations between the identified risk factors and the molecular subtype of the tumor. All statistical tests were two-sided with a pre-determined cutoff for statistical significance at alpha = 0.05. The computer software Stata, version 14, was used for all statistical analyses.

## Results

The proportions of tumors larger than 2 cm at diagnosis were 19% (*n* = 281) and 34% (*n* = 185) for screen-detected and interval cancers, respectively (Table 1). Table 2 shows that, of all examined factors, only BMI and PD were associated with having a tumor larger than 2 cm. When stratifying by length of delay between diagnosis and BMI ascertainment, the odds ratios for the associations between BMI and being diagnosed with a large cancer were 2.56, 2.34, and 2.23 in women with a delay of less than 3 years, from 3 years to less than 6 years, and 6 years or more, respectively.

**Table 1:** Description of study population, follow up, and tumor characteristics

	All	Screen-detected cancer	Interval cancer	Subtype-determined^a^
	*n* = 2012	*n* = 1466	*n* = 546	*n* = 478
Characteristic	Mean (SD) or proportion	Mean (SD) or proportion	Mean (SD) or proportion	Mean (SD) or proportion
Proportion large tumors (> 2 cm)	23%	19%	34%	27%
Proportion interval cancer	27%	0%	100%	24%
Age at diagnosis (years)	60.3 (5.4)	60.4 (5.4)	60.0 (5.5)	61.6 (5.4)
BMI (kg/m^2^)	25.6 (4.3)	26.0 (4.3)	24.7 (3.9)	26.4 (4.3)
BMI <25	47%	44%	57%	39%
BMI ≥25	53%	56%	43%	61%
PD (%)	20.5 (13.4)	18.9 (12.8)	24.9 (14.0)	20.6 (16.2)
PD <20	56%	61%	41%	57%
PD ≥20	44%	39%	59%	43%
Age at menarche (years)	13.2 (1.5)	13.2 (1.5)	13.3 (1.5)	13.1 (1.4)
Oral contraceptives (ever)	78%	77%	80%	78%
Nulliparous	15%	15%	16%	16%
Age at first birth (parous women only)	25.6 (5.1)	25.6 (5.2)	25.7 (5.0)	25.8 (5.4)
Hormone replacement therapy (ever)	63%	61%	70%	61%
Family history (yes)	19%	19%	21%	20%
Follow-up time (person-years)	22,669	16,603	6066	3990
Breast-cancer events^a^ (count)	149	80	69	31
Tumor size (mm)	16.9 (11.7)	15.8 (11.3)	19.6 (12.3)	17.6 (11.8)
Histological type				
Ductal invasive	70%	70%	71%	78%
Lobular invasive	14%	14%	15%	13%
Other invasive	15%	16%	14%	9%
Estrogen receptor positive	88%	91%	79%	88%
Molecular subtype, surrogate^b^				
Luminal A	–	–	–	45%
Luminal B	–	–	–	42%
Her2-overexpressing	–	–	–	5%
Basal-like	–	–	–	8%

*BMI* body mass index; *PD* percent density, *Her2* human epidermal growth factor receptor 2

^a^Defined as the first of local recurrence, distant metastasis, or breast-cancer-specific death during follow up after initial diagnosis

^b^Subtype-determined group includes only women for whom complete data on tumor receptor status, Ki-67, PD, and BMI were available

**Table 2:** Associations between risk factors and having a tumor larger than 2 cm at diagnosis, estimated by logistic regression modeling

	Odds ratio (95% CI) for having a large vs. small tumor^a^	
	*n* = 1931	
Risk factor	Crude	Multiple adjusted^b^
Age at diagnosis (OR per 10 years)	0.83 (0.68 to 1.01)	0.87 (0.71 to 1.05)
BMI ≥25 vs. BMI <25	**1.34 (1.08 to 1.66)**	**1.47 (1.18 to 1.84)**
PD ≥20 vs. PD <20	**1.25 (1.01 to 1.54)**	**1.36 (1.09 to 1.70)**
Age at menarche (OR per year)	0.99 (0.92 to 1.06)	1.01 (0.94 to 1.09)
Oral contraceptives (ever/never)	1.09 (0.84 to 1.42)	1.06 (0.82 to 1.39)
Nulliparous (yes/no)	1.28 (0.96 to 1.70)	1.28 (0.97 to 1.71)
Age at first birth (OR per 10 years)	1.02 (0.81 to 1.28)	1.02 (0.81 to 1.28)
Hormone replacement therapy (ever/never)	1.01 (0.81 to 1.26)	1.06 (0.84 to 1.34)
Family history of breast cancer (yes/no)	0.97 (0.74 to 1.28)	0.98 (0.74 to 1.29)

Values in bold represent associations with *p* values <0.05

*BMI* body mass index, *PD* mammographic percent density

^a^Outcome: large is > 2 cm, small is ≤ 2 cm

^b^Adjusted for age at diagnosis, BMI and PD. All women with complete data on these variables were included

The left side of Table 3 shows the results of the multiple adjusted logistic regression modeling, stratified by detection mode, with dichotomized tumor size as the outcome. High BMI was associated with having a large screen-detected tumor at diagnosis (OR 1.37; 95% CI 1.02 to 1.83) or a large interval tumor (OR 2.12; 95% CI 1.41 to 3.18). Introducing a term for the time between diagnosis and study entry caused minimal change in the estimated ORs related to high BMI; for screen-detected cancers the OR changed from 1.37 (as stated previously) to 1.32 (95% CI 0.98 to 1.77) and for interval cancers the OR changed from 2.12 (as stated previously) to 2.10 (95% CI 1.39 to 3.15). High PD was positively associated with tumor size in screen-detected cancers at diagnosis (OR 1.72; 95% CI 1.29 to 2.30) and negatively associated with tumor size in interval cancers (OR 0.60; 95% CI 0.40 to 0.90). The effect of BMI on tumor size was similar when examining the groups of women with low and with high PD separately, with OR 1.80 (95% CI 1.02 to 3.17) and OR 2.26 (95% CI 1.34 to 3.8), respectively. Introducing a term for the time between diagnosis and study entry caused minimal change in the estimated ORs related to high PD; for screen-detected cancers the OR changed from 1.74 (as stated previously) to 1.71 (95% CI 1.28 to 2.29) and for interval cancers the OR changed from 0.62 (as stated previously) to 0.59 (95% CI 0.39 to 0.90).

**Table 3:** Association between identified risk factors and tumor size for each mode of detection, estimated by logistic regression with tumor size ≤2 cm or >2 cm as outcome and by linear regression with tumor size in millimeters as outcome

		Odds ratio (95% CI) for having a large vs. small tumor^a^		β = Linear regression coefficient (95% CI)	
Detection mode	Number	BMI ≥25 vs. BMI <25	PD ≥20 vs. PD <20	BMI ≥25 vs. BMI <25	PD ≥20 vs. PD <20
All	1932	**1.47 (1.18 to 1.84)**	**1.36 (1.09 to 1.70)**	**1.8 (0.7 to 2.9)**	**1.4 (0.3 to 2.6)**
Screen-detected cancer	1402	**1.34 (1.01 to 1.78)**	**1.75 (1.32 to 2.32)**	1.0 (−0.2 to 2.3)	**2.4 (1.2 to 3.7)**
Interval cancer	530	**2.03 (1.39 to 2.99)**	**0.62 (0.42 to 0.92)**	**4.6 (2.4 to 6.8)**	**−2.9 (−5.2 to − 0.7)**

Adjusted for age at diagnosis, body mass index (BMI), and mammographic percent density (PD). All women with complete data on these risk factors were included in the analysis. Values in bold represent associations with *p* values <0.05

^a^Outcome: large is > 2 cm, small is ≤ 2 cm

The right side of Table 3 shows the results of the multiple adjusted linear regression modeling, stratified by detection mode, with continuous tumor size as the outcome. The coefficients for BMI were 1.0 mm (95% CI −0.3 to 2.2) and 4.8 mm (95% CI 2.6 to 7.1) for screen-detected and interval cancers, respectively. For PD, the coefficients were 2.2 mm (95% CI 0.9 to 3.5) for screen-detected cancers, and −3.2 mm (95% CI −5.4 to −0.9) for interval cancers.

In Additional file 2: Figure S1, the graphs show the model-predicted probabilities of having a tumor larger than 2 cm as a function of continuous measures of BMI and PD, keeping all other covariates at their mean value, stratified by detection mode. For women with interval cancer, the probability of a large tumor increased markedly with increasing BMI.

**Fig. 1:**
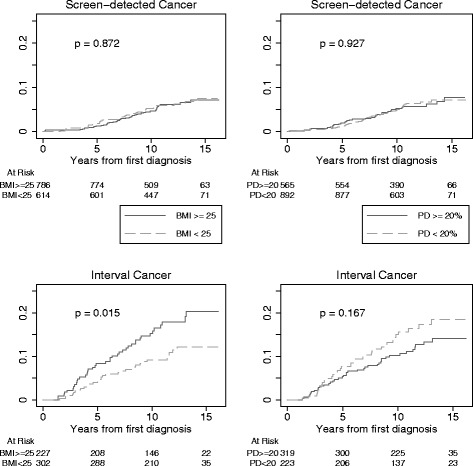
Kaplan-Meier plot of the cumulative hazard rate for breast-cancer event (first event of loco-regional recurrence, distant metastasis, or death caused by breast cancer) for body mass index (BMI) to the left and mammographic percent density (PD) to the right, plotted separately for each detection mode. Log-rank test *p* value is shown in each graph

Figure 1 shows the Kaplan-Meier cumulative hazard rate plot for breast-cancer events in relation to PD and BMI. Among women with interval breast cancer, prognosis was worse in those with high compared to low BMI (log-rank test *p* value = 0.015). The association remained significant after multiple adjusted Cox regression (Table 4). Among women with interval cancer, the hazard ratio (HR) for high vs. low BMI was 1.70 (95% CI 1.04 to 2.77) after multiple adjustments. The increased risk of breast-cancer events among women with interval cancers when comparing high to low BMI was similar when examining the groups of women with low and with high PD separately, with HR 1.82 (95% CI 0.85 to 3.90) and HR 1.64 (95% CI 0.85 to 3.14), respectively. There was no statistically significant association between breast-cancer event and BMI among women with screen-detected cancers. There was no association between breast-cancer event and PD in any detection mode. Adding the terms of year of diagnosis (*p* = 0.50) and the time between diagnosis and study entry (*p* = 0.43) suggested no influence of survival bias related to timing of study entry. An additional sensitivity analysis was performed by limiting the survival analysis to the 20% of cases diagnosed closest to study entry. For these women with a short time between diagnosis and study inclusion the point estimate of the HR for the association between BMI and the breast-cancer event was higher (2.20; 95% CI 0.61 to 7.98).

**Table 4:** Survival analysis: associations between patient characteristics and disease progression (first event of loco-regional relapse, distant metastasis, or death due to breast cancer), estimated by Cox regression

				Hazard ratio (95% CI)	
Detection mode	Number	Person-years	Events	Events BMI ≥25 vs. BMI <25	PD ≥20 vs. PD <20
All	1925	21,859	153	1.18 (0.84 to 1.64)	1.11 (0.79 to 1.55)
Screen-detected cancers	1397	15,932	81	0.97 (0.62 to 1.52)	0.99 (0.62 to 1.58)
Interval cancers	528	5927	72	**1.70 (1.04 to 2.77)**	1.03 (0.57 to 1.53)

Follow up ended on 31 December 2015. The hazard ratio estimates were adjusted for age at diagnosis, body mass index (BMI) and mammographic percent density (PD). Values in bold represent associations with *p* values <0.05

**Fig. 2:**
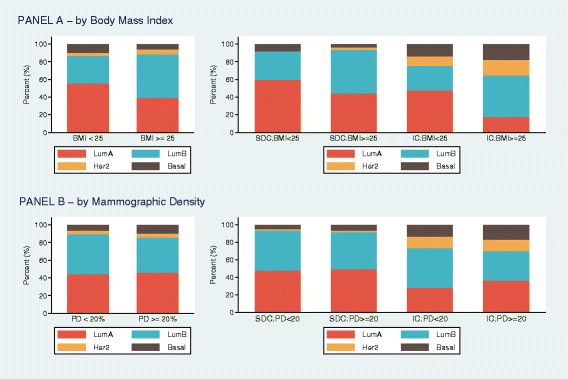
**a** Distribution of molecular subtype by each combination of detection mode and body mass index (BMI) (low < 25 kg/m2, high > = 25 kg/m2). **b** Distribution of molecular subtype by each combination of detection mode and percent density (PD) (low < 20%, high > = 20%). SDC, screen-detected cancer; IC, interval cancer, LumA, luminal A; LumB, luminal; Her2, human epidermal growth factor receptor 2. Chi-square test *p* values are shown comparing subtype distributions between women with high and low BMI or PD, within each detection mode

Figure 2 shows the distribution of molecular subtypes taking into account high or low BMI or PD and the detection mode. Regardless of the detection mode, BMI influenced the distribution of molecular subtypes. The findings were confirmed after multiple adjusted logistic regression modeling (Additional file 1: Table S2). High BMI was associated with an increased proportion of luminal B compared to luminal A breast cancer, overall (RRR 2.13; 95% CI 1.33 to 3.40) as well as within each detection mode. In addition, among women with interval cancer, high BMI was associated with an increased proportion of Her2-overexpressing (RRR 17.7; 95% CI 2.46 to 128) and basal-like cancers (RRR 7.68; 95% CI 1.39 to 42.5), compared to luminal A cancer. PD was not associated with the molecular subtype, either overall or within the detection mode subgroups.

## Discussion

In summary, we found that women with high compared to low BMI more often had large tumors and an increased risk of an aggressive molecular subtype. Both these findings were more pronounced among interval cancers. Among women with interval cancers, those with high BMI had a worse long-term prognosis. Having high PD was associated with having large tumors at screen detection, but with small tumors among women with interval cancer. PD was not associated with prognosis or molecular subtype. In agreement with a previous paper from our group by Holm et al. [6], the current manuscript shows (descriptively in Table 1) that the proportion of tumors larger than 2 cm was higher among interval cancers (34%) compared to screen-detected cancers (19%). The study by Holm et al. was primarily focused on interval cancer – attempting to understand how risk factors and tumor characteristics differed between women with high compared to low mammographic density. The current manuscript is primarily focused on large cancers – attempting to understand how risk factors and tumor characteristics differ by detection mode, with the aim of being useful for modifying screening programs. Additionally, long-term follow up was performed based on the identified risk factors to examine whether it would be likely that focusing on those risk factors could affect prognosis.

That women with high BMI have larger tumors at diagnosis is supported by previous studies [15, 16], but those were not stratified by detection mode. We found that among women with interval cancer, having high BMI doubled the risk of being diagnosed with a large tumor while among women with screen-detected cancer the risk increase was less pronounced. Based on published findings from our group, we speculate that high BMI makes clinical detection especially difficult, but has limited or no effect on mammographic detection [17]. The clinical difficulty is possibly related to women with higher BMI, on average, having a larger breast size, which reduces the sensitivity of palpation. In a Norwegian study performed by Maehle et al., which included breast cancer cases diagnosed before public screening mammography was introduced, it was observed that there was a significant difference in BMI (measured on average 12.5 years before diagnosis) between women diagnosed with tumors smaller than 2 cm and women diagnosed with tumors larger than 2 cm [18]. An additional hypothesis is that BMI is related to faster-growing tumors since it has been shown that tumors in women with high BMI were more likely to express markers of high proliferation [19]. The biological mechanism has been suggested to be that women with high BMI have a higher local aromatase gene expression resulting in higher local estrogen levels [20].

Our results cast new light on the finding in the paper mentioned in the "Background" section, that the incidence of large tumors had not decreased after the introduction of breast cancer screening programs, when comparing the late 1970s with the late 2000s [2]. The American NHANES surveys found that the proportion of obese women was 17.0% in 1976 to 1980, and had increased to 36.1% in 2009 to 2010 [21]. Given our finding that higher BMI is strongly associated with larger tumor size at clinical detection, it is possible that the incidence of large tumors would have been markedly higher today without the establishment of mammographic screening programs. Another important consideration is that our results based on women regularly attending screening suggest that it is especially important for women with high BMI to attend screening. There is mixed evidence from prior studies on the current attendance rate; in some studies women with high BMI were less likely to attend than women with normal BMI while in others there was no significant difference [22, 23].

In contrast, even though women with high PD more often are diagnosed with interval cancer, among women with interval cancer the tumors were smaller in women with high PD than in women with low PD. The basis for this observation might be that the meaning of a negative screening mammogram differs between dense and less dense breasts. In less dense breasts, the majority of tumors are detected. In dense breasts, only the tumors that have reached a larger size since the last screening are detected while the smaller, presumably slower-growing ones, to a larger extent remain undetected. This hypothesis is supported by a prior study from our group, where it was shown that interval cancers in breasts with high PD were not more aggressive than screen-detected cancers [6]. The increased likelihood of detecting the tumor whilst it is still small might be related to the slower growth rate.

Breast-cancer event, defined as the first event of loco-regional recurrence, distant metastasis, or death due to breast cancer, was associated with BMI among women with interval cancer. No such association was identified for women with screen-detected cancer. Previous studies have not taken detection mode into account. Some have demonstrated that high BMI is associated with a worse prognosis [19, 24–27], while other studies have not [28–31]. Our results, showing that BMI adversely affects prognosis among clinically detected cancers only, are in agreement with a prior Swedish study suggesting that the prognostic effect of BMI was stronger before than after the introduction of population-wide screening programs [32]. The association between BMI and prognosis might be related both to factors at diagnosis, i.e., larger tumors, but also to other factors related to BMI after taking both stage and treatment into account, as was shown by Gierach et al. [33] in a prior study of over 9000 patients with breast cancer in the US Breast Cancer Surveillance Consortium. In the same study high mammographic density was not associated with mortality, which is in line with our not finding an association between PD and prognosis both in screen-detected and interval cancers.

To some extent, the adverse effect on breast cancer prognosis might be explained by our finding that women with high BMI had tumor molecular subtypes that are known to be associated with poor prognosis [34]. The associations found regarding molecular subtypes of interval cancers were based on a relatively small sample. Therefore, one should be cautious about making any clear conclusions from these findings. In our data, among both women with interval cancers and screen-detected cancers, high BMI was associated with a relative increase in the luminal B. However, high BMI was associated with a relative increase in basal-like and Her2-overexpressing tumors only in women with interval cancer. The instability of receptor expression throughout breast cancer tumor progression has been demonstrated previously [35]. A speculative hypothesis on a biological basis could be that women with high BMI have tumors that from the start have a higher proliferation rate, and that such tumors that left undiagnosed for longer due to difficulty in palpation have more opportunity to transition into Her2 overexpressing or basal-like subtypes. The lack of association between PD and molecular subtype, even after stratification by mode of detection, can be due to low statistical power or it can be due to PD being a marker of general breast cancer risk rather than subtype-specific risks. This is in line with prior case-case studies in which PD did not differ between molecular subtypes, and cohort studies showing similarly increased risks irrespective of the molecular subtype [36–38].

A major strength of our study is that it was performed within a population-based screening cohort. Further, the study had completeness of follow up during an extensive time period, up to 14 years after diagnosis. A weakness of our study was that mammographic percent density was estimated on mammograms between 3 years before and up until diagnosis. However, we only used contralateral mammograms to ensure that density was not influenced by tumor presence. A potential weakness is that BMI was measured at study inclusion after diagnosis. This opens up the theoretical possibility of reverse causality, i.e., a larger tumor might cause a rise in BMI. However, this does not seem biologically plausible. Our sensitivity analysis of the risk associations between BMI and tumor size that was stratified by the time between diagnosis and BMI measurement showed similar effect sizes of the association independent of this time delay. Furthermore, meta-analysis has shown that higher BMI is associated with worse prognosis regardless of the time when it is ascertained, i.e., if it was before or after diagnosis, or if it was more or less than 12 months after diagnosis [25]. Another potential weakness concerns the observed differences in weight gain related to differences in chemotherapy and chemo-hormonal therapy [39]. Adjusting the models for differences in the use of adjuvant treatment between different cancer subtypes did not change our results. However, such an approach might not fully account for treatment effects. Finally, having a categorical cutoff at a certain tumor size is to some extent arbitrary, even if the same limit has been used in several other publications and systems. Therefore, it was reassuring that our linear regression modeling confirmed the corresponding associations with BMI and PD. A limitation was the relatively small number of tumors for which the molecular surrogate subtypes could be determined, which means that the estimated association measures for tumor subgroups have high uncertainty.

When considering changes to the current screening paradigm, it is important to take absolute risks into account. Our study population of cases did not allow a direct calculation of absolute risks. However, an analysis by Törnberg et al. [4] of Swedish screening examinations among women regularly attending screening between 1989 and 1997 showed that the absolute incidence per 10,000 screenings was 22 and 48 for interval cancer and screen-detected cancer, respectively. Törnberg et al. [4] also showed that among the interval cancers with available staging information, 30% were stage T2 or higher (corresponding to a tumor size of more than 2 cm). The corresponding percentage of large cancers in our study was 34%, which is in line with this prior report. The absolute incidence of large interval cancers can be estimated to be 7 per 10,000 screenings. Combining the incidence rate in the prior report with the proportions in our study, the absolute incidence of large screen-detected tumors would similarly be estimated to be 9 per 10,000 screenings. Answering the question as to whether or not making changes to the screening program to avoid some of these large tumors would have a beneficial cost-effectiveness ratio is beyond the scope of our current study.

## Conclusions

In conclusion, women with high BMI had an increased risk of having a tumor larger than 2 cm at diagnosis, and had a worse long-term prognosis if their tumor was detected as an interval cancer. Therefore, women with high BMI should be especially encouraged to participate in screening. To study the effects of potential changes to current screening programs, we propose that high PD and high BMI could independently trigger an adapted screening regime, for example, high PD could trigger adjunct screening modalities such as ultrasound or magnetic resonance imaging (MRI), and high BMI could trigger more frequent screenings. Before implementing any such changes cost-effectiveness analyses, taking absolute risks into account, would need to be performed.

## Additional files

Additional file 1: **Table S1.** Linear regression coefficients for association between the tumor size (mm) and selected patient characteristics, multiple adjusted models. **Table S2.** Associations between patient characteristics and molecular subtype, overall and by each detection mode, estimated by multinomial logistic regression modeling. (DOCX 115 kb)

Additional file 2: **Figure S1.** Model-predicted probabilities of a large (as opposed to small) tumor as a function of BMI and PD, respectively, keeping all other covariates at their mean value. The probabilities were estimated separately for screen-detected cancers and interval cancers (clinically detected). The gray area around the curve corresponds to the 95% confidence intervals. (EPS 274 kb)

## Abbreviations

BMI: Body mass index
CI: Confidence interval
ER: Estrogen receptor
HER2: Human epidermal growth factor-2
HR: Hazard ratio
OR: Odds ratio
PD: Mammographic percent density
PR: Progesterone receptor
RRR: Relative risk ratio
SNP: Single nucleotide polymorphism

## References

[1] Lauby-Secretan B, Scoccianti C, Loomis D, Benbrahim-Tallaa L, Bouvard V, Bianchini F, Straif K (2015). Breast-cancer screening — viewpoint of the IARC Working Group. N Engl J Med.

[2] Welch HG, Prorok PC, O'Malley AJ, Kramer BS (2016). Breast-cancer tumor size, overdiagnosis, and mammography screening effectiveness. N Engl J Med.

[3] Carter CL, Allen C, Henson DE (1989). Relation of tumor size, lymph node status, and survival in 24,740 breast cancer cases. Cancer.

[4] Tornberg S, Kemetli L, Ascunce N, Hofvind S, Anttila A, Seradour B, Paci E, Guldenfels C, Azavedo E, Frigerio A (2010). A pooled analysis of interval cancer rates in six European countries. Eur J Cancer Prev.

[5] Barton MB, Elmore JG, Fletcher SW (1999). Breast symptoms among women enrolled in a health maintenance organization: frequency, evaluation, and outcome. Ann Intern Med.

[6] Holm J, Humphreys K, Li J, Ploner A, Cheddad A, Eriksson M, Tornberg S, Hall P, Czene K (2015). Risk factors and tumor characteristics of interval cancers by mammographic density. J Clin Oncol.

[7] Lind H, Svane G, Kemetli L, Tornberg S (2010). Breast Cancer Screening Program in Stockholm County, Sweden - aspects of organization and quality assurance. Breast care (Basel, Switzerland).

[8] Edge SB, Compton CC (2010). The American Joint Committee on Cancer: the 7th edition of the AJCC cancer staging manual and the future of TNM. Ann Surg Oncol.

[9] Li J, Szekely L, Eriksson L, Heddson B, Sundbom A, Czene K, Hall P, Humphreys K (2012). High-throughput mammographic-density measurement: a tool for risk prediction of breast cancer. Breast Cancer Res.

[10] Byng JW, Boyd NF, Fishell E, Jong RA, Yaffe MJ (1994). The quantitative analysis of mammographic densities. Phys Med Biol.

[11] Goldhirsch A, Winer EP, Coates AS, Gelber RD, Piccart-Gebhart M, Thürlimann B, Senn HJ, Panel M, Albain KS, André F (2013). Personalizing the treatment of women with early breast cancer: highlights of the St Gallen International Expert Consensus on the Primary Therapy of Early Breast Cancer 2013. Ann Oncol.

[12] Mavaddat N, Pharoah PD, Michailidou K, Tyrer J, Brook MN, Bolla MK, Wang Q, Dennis J, Dunning AM, Shah M *et al*: Prediction of breast cancer risk based on profiling with common genetic variants. J Natl Cancer Inst. 2015;107(5);djv036. 10.1093/jnci/djv036 PMC4754625 25855707

[13] Manual for national quality registry for breast cancer/follow-up [http://cancercentrum.se/globalassets/cancerdiagnoser/brost/kvalitetsregister/manualuppfoljning-ver-1.0.3.pdf]. Accessed 8 Jan 2017.

[14] Johansson LA, Westerling R (2000). Comparing Swedish hospital discharge records with death certificates: implications for mortality statistics. Int J Epidemiol.

[15] Robinson B, Currie M, Phillips E, Dachs G, Strother M, Morrin H, Davey V, Frampton C (2017). Body mass index (BMI): association with clinicopathological factors and outcome of women with newly diagnosed breast cancer in New Zealand. N Z Med J.

[16] Carmichael AR, Bates T (2004). Obesity and breast cancer: a review of the literature. Breast (Edinburgh, Scotland).

[17] Abrahamsson L, Czene K, Hall P, Humphreys K (2015). Breast cancer tumour growth modelling for studying the association of body size with tumour growth rate and symptomatic detection using case-control data. Breast Cancer Res.

[18] Maehle BO, Tretli S, Skjaerven R, Thorsen T (2001). Premorbid body weight and its relations to primary tumour diameter in breast cancer patients; its dependence on estrogen and progesteron receptor status. Breast Cancer Res Treat.

[19] Daling JR, Malone KE, Doody DR, Johnson LG, Gralow JR, Porter PL (2001). Relation of body mass index to tumor markers and survival among young women with invasive ductal breast carcinoma. Cancer.

[20] Bulun SE, Mahendroo MS, Simpson ER (1994). Aromatase gene expression in adipose tissue: relationship to breast cancer. J Steroid Biochem Mol Biol.

[21] Prevalence of overweight, obesity, and extreme obesity among adults: United States, trends 1960-62 through 2009-2010 [https://www.cdc.gov/nchs/data/hestat/obesity_adult_09_10/obesity_adult_09_10.htm]. Accessed 8 Jan 2017.

[22] Atkins E, Madhavan S, LeMasters T, Vyas A, Gainor SJ, Remick S (2013). Are obese women more likely to participate in a mobile mammography program?. J Community Health.

[23] Eichholzer M, Richard A, Rohrmann S, Schmid S, Guth U (2016). Overweight, obesity, and breast cancer screening: results from the 2012 Swiss Health Survey. Eur J Cancer Prev.

[24] Berclaz G, Li S, Price KN, Coates AS, Castiglione-Gertsch M, Rudenstam CM, Holmberg SB, Lindtner J, Erien D, Collins J (2004). Body mass index as a prognostic feature in operable breast cancer: the International Breast Cancer Study Group experience. Ann Oncol.

[25] Chan DSM, Vieira AR, Aune D, Bandera EV, Greenwood DC, McTiernan A, Navarro Rosenblatt D, Thune I, Vieira R, Norat T (2014). Body mass index and survival in women with breast cancer—systematic literature review and meta-analysis of 82 follow-up studies. Ann Oncol.

[26] Goodwin PJ, Boyd NF (1990). Body size and breast cancer prognosis: a critical review of the evidence. Breast Cancer Res Treat.

[27] Ewertz M, Jensen MB, Gunnarsdottir KA, Hojris I, Jakobsen EH, Nielsen D, Stenbygaard LE, Tange UB, Cold S (2011). Effect of obesity on prognosis after early-stage breast cancer. J Clin Oncol.

[28] Dignam JJ, Wieand K, Johnson KA, Fisher B, Xu L, Mamounas EP (2003). Obesity, tamoxifen use, and outcomes in women with estrogen receptor-positive early-stage breast cancer. J Natl Cancer Inst.

[29] den Tonkelaar I, de Waard F, Seidell JC, Fracheboud J (1995). Obesity and subcutaneous fat patterning in relation to survival of postmenopausal breast cancer patients participating in the DOM-project. Breast Cancer Res Treat.

[30] Carmichael AR, Bendall S, Lockerbie L, Prescott RJ, Bates T (2004). Does obesity compromise survival in women with breast cancer?. Breast (Edinburgh, Scotland).

[31] Sohrabi A, Sandoz J, Spratt JS, Polk HC (1980). Recurrence of breast cancer. Obesity, tumor size, and axillary lymph node metastases. JAMA.

[32] Olsson A, Garne JP, Tengrup I, Zackrisson S, Manjer J (2009). Body mass index and breast cancer survival in relation to the introduction of mammographic screening. Eur J Surg Oncol.

[33] Gierach GL, Ichikawa L, Kerlikowske K, Brinton LA, Farhat GN, Vacek PM, Weaver DL, Schairer C, Taplin SH, Sherman ME (2012). Relationship between mammographic density and breast cancer death in the Breast Cancer Surveillance Consortium. J Natl Cancer Inst.

[34] Sørlie T, Perou CM, Tibshirani R, Aas T, Geisler S, Johnsen H, Hastie T, Eisen MB, van de Rijn M, Jeffrey SS (2001). Gene expression patterns of breast carcinomas distinguish tumor subclasses with clinical implications. Proc Natl Acad Sci.

[35] Lindström LS, Karlsson E, Wilking UM, Johansson U, Hartman J, Lidbrink EK, Hatschek T, Skoog L, Bergh J (2012). Clinically used breast cancer markers such as estrogen receptor, progesterone receptor, and human epidermal growth factor receptor 2 are unstable throughout tumor progression. J Clin Oncol.

[36] Sartor H, Zackrisson S, Elebro K, Hartman L, Borgquist S (2015). Mammographic density in relation to tumor biomarkers, molecular subtypes, and mode of detection in breast cancer. Cancer Causes Control.

[37] Ma H, Luo J, Press M, Wang Y, Bernstein L, Ursin G (2009). Is there a difference in the association between percent mammographic density and subtypes of breast cancer? Luminal A and triple-negative breast cancer. Cancer Epidemiol Biomarkers Prev.

[38] Eriksson L, Hall P, Czene K, Dos Santos S, McCormack V, Bergh J, Bjohle J, Ploner A (2012). Mammographic density and molecular subtypes of breast cancer. Br J Cancer.

[39] Camoriano JK, Loprinzi CL, Ingle JN, Therneau TM, Krook JE, Veeder MH (1990). Weight change in women treated with adjuvant therapy or observed following mastectomy for node-positive breast cancer. J Clin Oncol.

