# Supplementary material for: Long-term prognostic implications of risk factors associated with tumor size: a case study of women regularly attending screening
Source: Breast Cancer Res. 2018 Apr 18;20:31. doi: 10.1186/s13058-018-0962-6 (PMC5907386; doi:10.1186/s13058-018-0962-6)
Supplement: Supplementary file 1 [file 13058_2018_962_MOESM1_ESM.docx]

Supplemental Table S1. Linear regression coefficients for association between the tumor size (mm) and selected patient characteristics, multiple adjusted models.

	β = Linear regression coefficient (95% CI)					
Predictor	Overall (*n* = 1,809)		Screen-detected cancer (*n* = 1,299)		Interval cancer (*n* = 510)	
Age at Diagnosis (mm per 5 years)	-0.12	(-0.64 to 0.40)	0.17	(-0.43 to 0.77)	-0.70	(-1.67 to 0.27)
BMI (mm per 5 kg/m2)	**1.33**	**(0.62 to 2.03)**	**1.13**	**(0.35 to 1.91)**	**2.73**	**(1.27 to 4.19)**
Mammographic Percent Density (mm per 10%)	**0.61**	**(0.16 to 1.05)**	**1.14**	**(0.61 to 1.67)**	**-1.11**	**(-1.92 to -0.31)**

Only women with complete data on all characteristics were included in the analyses

Numbers in bold represent associations with a p-value below 0.05

Supplemental Table S2. Associations between patient characteristics and molecular subtype, overall and by each detection mode, estimated by multinomial logistic regression modeling.

Detection mode and Molecular Subtype	*n*	Relative Risk Ratio (95% CI) BMI ≥ 25 vs. BMI < 25		PD ≥ 20 vs. PD < 20	
All					
Luminal A	206	Base outcome		Base outcome	
Luminal B	191	**2.25**	**(1.44 to 3.51)**	0.97	(0.62 to 1.51)
Her2-overexpressing	21	2.62	(0.92 to 7.41)	1.47	(0.56 to 3.87)
Basal-like	35	0.99	(0.46 to 2.12)	1.50	(0.69 to 3.24)
Screen-detected cancers					
Luminal A	170	Base outcome		Base outcome	
Luminal B	151	**2.02**	**(1.22 to 3.34)**	0.96	(0.59 to 1.57)
Her2-overexpressing	6	NA		NA	
Basal-like	18	0.63	(0.22 to 1.76)	1.36	(0.48 to 3.82)
Interval cancers					
Luminal A	36	Base outcome		Base outcome	
Luminal B	40	**4.42**	**(1.50 to 13.1)**	0.92	(0.32 to 2.65)
Her2-overexpressing	15	**5.33**	**(1.28 to 22.2)**	1.36	(0.34 to 5.49)
Basal-like	17	**4.18**	**(1.08 to 16.2)**	1.56	(0.41 to 5.97)

BMI = Body Mass Index. PD = Mammographic percent density. NA = Not applicable to estimate, too few observations
The relative risk ratio estimates are adjusted for age at diagnosis, BMI and PD.
Numbers in bold represent associations with a p-value below 0.05
